# Cognitive decline is related to high blood glucose levels in older Chinese adults with the ApoE ε3/ε3 genotype

**DOI:** 10.1186/s40035-019-0151-2

**Published:** 2019-04-03

**Authors:** Qi Qiu, Xiang Lin, Lin Sun, Min-jie Zhu, Tao Wang, Jing-hua Wang, Guan-jun Li, Shi-fu Xiao, Xia Li

**Affiliations:** Department of Psychogeriatrics, Shanghai Mental Health Center, Shanghai Jiao Tong University School of Medicine, South WanPing Road 600, Shanghai, 200030 China

**Keywords:** Apolipoproteins E, Cognition, Blood glucose, Aged, White matter, Community-based

## Abstract

**Background:**

Few studies have investigated the effects of blood glucose (BG) on cognitive function in community-dwelling elderly individuals carrying the apolipoprotein E (APOE) ε3 allele.

**Objective:**

To explore the effect of high BG levels on cognitive function in APOE ε3-carrying, non-demented, community-dwelling older adults, as compared to their counterparts carrying the APOE ε4 or APOE ε2 alleles.

**Methods:**

Within the China Longitudinal Ageing Study, we recruited 282 elderly adults without dementia. Data collected included demographic information; psychological measures; laboratory test results, including BG and plasma lipid levels; and APOE genotypes. We divided the participants into APOE ε2(ε2/ε2, ε2/ε3), ε3(ε3/ε3), and ε4(ε3/ε4, ε4/ε4) groups. Partial correlation analyses and multivariate linear regression analyses were utilized to compare the cognitive function and laboratory data between the APOE groups. White matter hyperintensity (WMH) was measured on magnetic resonance images in 77 participants.

**Results:**

With adjustment for age, sex, education, and diabetes, higher BG in non-demented community-dwelling older adults was associated with cognitive decline in immediate memory and executive function. In the APOE ε3 group, elevated BG was associated with cognitive decline in immediate memory, executive function, and perceptual reasoning. In the APOE ε4 group, higher BG was also correlated with a decline in abstract reasoning. There was a trend for association between higher BG and more severe WMHs.

**Conclusion:**

Worse cognitive function was correlated withApoEε3/ε3 genotype carriers with higher BG in community-dwelling older adults.

## Background

As the world population ages, the number of people living with cognitive disease is rising rapidly. The World Alzheimer Report 2018 reports that the number of people living with dementia is likely to triple from 50 million to 152 million by 2050 [1]. Most of the affected individuals are from middle and lower income countries [1]. The associated cognitive impairment leads to a high rate of disability and generates a heavy economic burden.

Many clinical and population-based studies suggest cognitive function is associated with high blood levels of total cholesterol (TC), low-density lipoprotein cholesterol (LDL-C), triglycerides (TG), glucose, and low levels of high -density lipoprotein cholesterol (HDL-C) [2, 3]. Gill Livingston and her colleagues have suggested that diabetes is a modifiable risk factor of dementia [4]. A study that investigated the Chinese Alzheimer’s disease (AD) population found that high normal fasting blood glucose (BG) level was associated with dementia, independently of vascular risk factors [5]. Moreover, among dementia-free older adults, those with high BG also had poorer overall cognitive performance [6]. The toxicity of hyperglycemia might mediate microvascular abnormalities and may be associated with greater white matter lesion volumes [7, 8].

A well-established susceptibility genetic factor for Alzheimer’s disease is the apolipoprotein E (APOE) ε4 allele [9]. APOE in the central nervous system is key to the uptake of lipids and their reallocation to cells for myelin generation or membrane repair [10]. APOE ε4 preferentially combines large lipoprotein particles and is associated with an increased risk for atherosclerosis [11] and vascular dementia [12]. APOE has been shown to stimulate Aβ production in human neurons with an APOE 4 > APOE3 > APOE2 potency rank order [13]. Some drugs affect cholesterol metabolism through mechanisms that probably induce APOE synthesis [14]. In peripheral blood studies, APOE ε4 was shown to be associated with higher total cholesterol and higher LDL-C levels, whereas APOE ε2 was associated with lower levels of these markers [15]. But in our previous study, APOEε3/ε3 genotype carriers among community-dwelling elderly individuals exhibited higher BG [16]. Some researchers have reported that diabetes and the APOE ε4 allele interact to impact cognitive function [17]. Diabetes along with the APOE ε4 allele might cause neuronal and vessel damage, increasing the risk for AD or mixed dementias [18, 19]. APOE ε3/ε3 is the most frequent genotype and considered the neutral risk genotype. Few studies have investigated the correlation between cognitive function and BG or lipid profile in APOE ε3/ε3genotype carriers independently. Therefore, this study investigated the association between cognitive performance and both BG and the lipid profile in APOE ε3/ε3 genotype carriers, as compared to that in APOE ε4 or APOE ε2 allele carriers, in dementia-free, community-dwelling older Chinese Han adults.

## Methods

### Sample and design

This study involved random sampling of community-dwelling elderly individuals (over 60 years old) from Changning district and Pudong new district from 2011 to 2012. We included 283 participants without dementia. Twenty-one elderly individuals with dementia were excluded, among whom five participants were APOE ε4 carriers and fifteen were APOE ε3/ε3 carriers. The inclusion criteria were age of 60 years or older with normal audio-visual function after correction. Exclusion criteria were as follows: the participants had diagnosed dementia; a major psychiatric disorder, such as schizophrenia; or a systemic illness that affected their ability to complete the assessment.

### Procedures

This survey used the China Longitudinal Ageing Study (CLAS) protocol [20]. Data collected included general demographic information, standard psychometric scales, APOE genotyping, and laboratory test results.

APOE genotyping was based on allele-specific polymerase chain reaction (PCR) methodology adapted to real-time PCR using a TaqMan probe [21]. The laboratory tests included fasting BG, TC, TG, LDL-C, and HDL-C level assessments. Cognitive function was measured using the Beijing version of the Montreal Cognitive Assessment (MoCA) [22], and the Neuropsychological Test Battery (NTB) [23], administered by a psychologist. Attending-level psychiatrists collected information on the current state and history of disease, conducted physical examinations, and determined a diagnosis using the Structured Clinical Interview for DSM-IV (SCID).

Seventy-seven participants underwent magnetic resonance imaging (MRI) and white matter lesion hyperintensity (WMH) was evaluated using the Fazekas scale [24]. The scale is composed of two parts, the periventricular lesion hyperintensity (PWMH) and deep white matter lesion hyperintensity (DWMH). Each part was scored, with a score ranging from 0 to 3; thus, the total score ranged from 0 to 6, with a higher score indicating more serious lesions.

All the subjects had signed an informed consent at the start of the study, and ethical approval was obtained from Shanghai Mental Health Center.

### Cognitive function assessment

The Beijing version of MoCA is a brief cognitive screening tool for mild cognitive impairment. It includes executive function, attention, language, visuospatial and orientation, and was used to assess global cognitive function.

The NTB consists of the following seven components: 1) the Wechsler Memory-Digit Span (WMDS, score range 0 to 24), including the Digits Forward and Digits Backward section where participants repeat a forward and backward sequence of numbers, and each sequence of numbers has more digits than the last; 2) the Rey Auditory Verbal Learning-Immediate test (RAVL-I, 0 to 75), where participants are given a list of 15 unrelated words repeated over five different trials and are asked to repeat them; 3) the Wechsler Memory Visual-Immediate test (WMVis-I, 0 to 18), which includes functional and semantic connections (matching pictures belonging to one category), recognition (reconfirm whether the pictures appeared before or not), and visual matching and abstracting (select picture items according to certain rules); 4) the Controlled Word Association Test (COWAT) which assessed the spontaneous production of words belonging to the category “vegetable”, and combinations of words with the Chinese words “shui” and “fa”; 5) the Rey Auditory Verbal Learning Delayed test (RAVL-D, 0 to 45) in which another list of 15 unrelated words was given and the participants had to repeat the original list of 15 words, and then repeat them again after 30 min; 6) the Wechsler Adults Intelligence Scale - Picture Completion test (WAIS-PC, 0 to 21), in which participants had to specify certain absent portions of drawings of familiar items; and 7) the Wechsler Adults Intelligence Scale - Block Design (WAIS-BD, 0 to 48) where the participants used hand movements to rearrange blocks that had various color patterns on different sides to match a given pattern. The NTB was used to assess immediate memory (WMVis-I and RAVL-I), delayed memory (RAVL-D), executive function (WMDS and COWAT), and perceptual reasoning (WAIS-PC and WAIS-BD).

### Statistical analyses

The genotype groups were divided into APOE ε2 (APOE ε2/ε3 and ε2/ε2), APOE ε3 (ε3/ε3), and APOE ε4 (ε2/ε4, ε3/ε4, ε4/ε4). Comparisons in the three groups were performed with an analysis of variance for continuous variables, and post hoc analyses were done between each set of two groups. The χ2 test was used for dichotomous variables. BG, MoCA score, and HDL had non-normal distributions, so a non-parametric test was used. Partial correlation analyses were performed to evaluate associations among glucose, lipid profiles, and cognitive function, with adjustment for education, age, sex, and diabetes, in the different APOE groups. Dependent variables were the z-score of the MoCA and NTB scores. The independent variables were APOE genotype and laboratory test results. Multivariate linear regression analyses were used to examine the relationships between cognition, age, education, and laboratory data. As not all subjects underwent MRI (54 in the APOE ε3, 12 in the APOE ε4, and 11 in the APOE ε2 groups), we matched 12 participants in terms of age and sex between the normal (< 6.1 mmol/L) and abnormal (≥ 6.2 mmol/L) BG groups in the APOE ε3 group. We used the *t*-test to compare WMHs in these two BG groups, adjusting for education. Significance levels of 0.05 were used for all the tests. All statistical analyses were performed using SPSS version 21.0 (IBM Corp., Armonk, NY, USA).

## Results

### Demographic characteristics

This study was a cross-sectional study of CLAS and included 283 elderly people without dementia. The mean age at investigation was 71.63 years (± 8.21 years) and mean years of education was 8.78 (± 4.53); 169 (59.7%) participants were female, and 47 (16.6%) were APOE ε4 carriers. The mean MoCA score was 22.29 (5.77). Demographic information is shown in Table 1.

**Table 1:** Demographic information in the total sample and different APOE groups

	Total (*N* = 283)	APOE ε2 (*N* = 39)	APOE ε3 (*N* = 197)	APOE ε4 (*N* = 47)	ε2 vs ε3	ε2 vs ε4	ε3 vs ε4
					*p*		
Age (years)	71.63 (8.22)	71.64 (8.16)	72.14 (8.44)	69.45 (7.06)	0.727	0.217	0.044
Education (years)	8.78 (4.53)	8.13 (4.24)	8.51 (4.69)	10.43 (3.70)	0.625	0.019	0.009
BMI (kg/m^2^)	24.02 (3.36)	24.09 (3.35)	23.97 (3.39)	24.20 (3.28)	0.834	0.890	0.676
Female	169 (59.7%)	25 (64.1%)	118 (59.9%)	26 (55.3%)	0.707		
BG (mmol/L)	5.70 (2.00)	5.15 (0.98)	5.85 (2.23)	5.55 (1.44)	0.006	0.338	0.591
TC (mmol/L)	4.86 (1.10)	4.73 (1.10)	4.87 (1.14)	4.93 (0.95)	0.454	0.395	0.744
TG (mmol/L)	1.83 (1.31)	1.86 (0.92)	1.80 (1.25)	1.95 (1.74)	0.773	0.754	0.466
LDL-C (mmol/L)	2.90 (0.90)	2.57 (0.87)	2.94 (0.93)	3.03 (0.75)	0.020	0.018	0.516
HDL-C (mmol/L)	1.17 (0.27)	1.21 (0.32)	1.18 (0.27)	1.09 (0.22)	0.872	0.138	0.077
Hypertensive	151 (53.5%)	23 (60.5%)	104 (52.8%)	24 (51.15%)	0.636		
Diabetes mellitus	45 (15.3%)	3 (7.9%)	35 (17.8%)	7 (14.9%)	0.316		
Hyperlipidemia	52 (19.8%)	9 (23.7%)	36 (19.9%)	7 (15.9%)	0.084		
MoCA	22.29 (5.77)	22.10 (5.31)	21.94 (6.02)	23.91 (4.81)	0.998	0.281	0.055

*BMI* body mass index, *BG* blood glucose, *TC* total cholesterol, *TG* triglycerides, *LDL-C* low destiny lipoprotein cholesterol, *HDL-C* high destiny lipoprotein cholesterol, *MoCA* Montreal Cognitive Assessment

Data are shown as mean (SD) or number (percentage)

The participants in the APOE ε3 group were older than those in the APOE ε4 group (*p* = 0.044). Compared to those in the APOE ε3 and APOE ε4 groups, the individuals in the APOE ε2 group had less education (*p* = 0.019, *p* = 0.009, respectively). The BG in the APOE ε2 group was lower than that in the APOE ε3 group (*p* = 0.045). LDL-C levels in the APOE ε2 group were lower than in the APOE ε3 and APOE ε4 groups (*p* = 0.020, *p* = 0.018, respectively). The HDL-C in the APOE ε2 was higher than in the APOE ε4 group (*p* = 0.037). The MoCA scores and the current state and history of disease, including hypertension, diabetes mellitus, and hyperlipidemia, were not different among the APOE groups. Overall, 28 of 52 patients with hyperlipidemia and 36 of 45 people with diabetes were treated with drugs.

### Relationship between blood glucose and cognitive function

Six participants had missing NTB data. The partial correlation analysis between cognitive function and independent variables (glucose, lipid data) indicated that BG was related to RAVL-I, COWAT, RAVL-D scores, with adjustment for age, sex, education, and diabetes (Table 2). HDL was related to the WAIS-BD (*r* = 0.140, *p* = 0.025).

**Table 2:** Partial correlation between glucose and cognitive scores

	Blood glucose	
	*R*	*p*
MoCA	−0.064	0.285
WMDS	0.019	0.750
RAVL-I	−0.168	0.005
WMV is-I	−0.064	0.285
COWAT	−0.149	0.013
RAVL-D	−0.140	0.019
WAIS-PC	−0.086	0.155
WAIS-BD	−0.085	0.156

*MoCA* Montreal Cognitive Assessment, *WMDS* Wechsler Memory-Digit Span, *RAVL-I* Rey Auditory Verbal Learning-Immediate, *WMVis-I* Wechsler Memory Visual-Immediate, *COWAT* Controlled Word Association Test, *RAVL-D* Rey Auditory Verbal Learning-Delayed, *WAIS-PC* Wechsler Adults Intelligence Scale-Picture Completion, *WAIS-BD* Wechsler Adults Intelligence Scale-Block Design

**Fig. 1:**
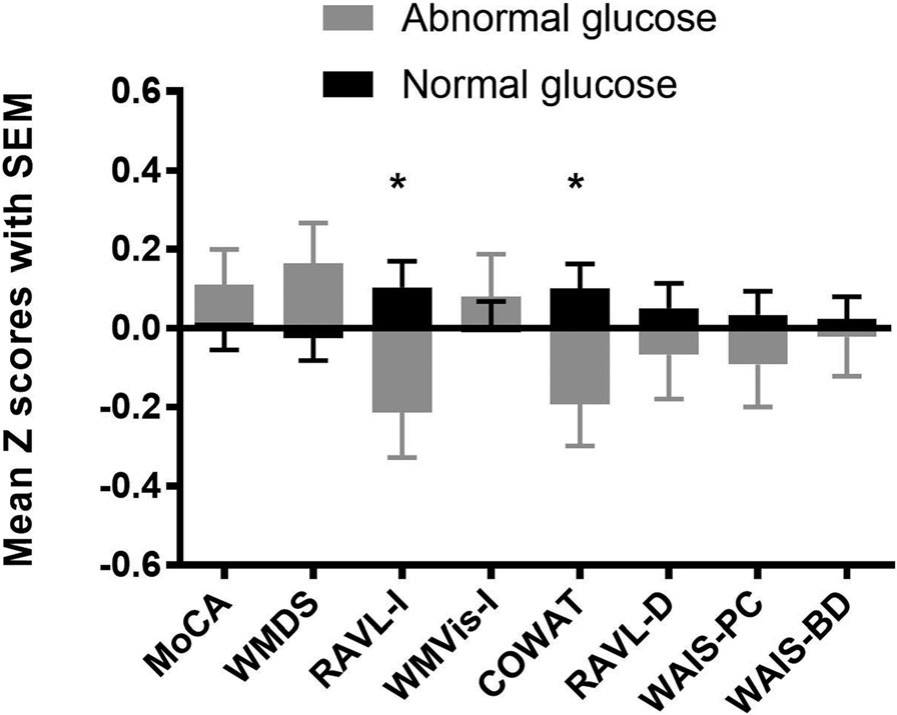
Differences in cognitive function between different BG groups, adjusting for age, sex, education, and diabetes. There were significant differences in the RAVL-I (*p* = 0.024) and COWAT (*p* = 0.022) scores between the abnormal and normal glucose groups. * *p* < 0.05

Seventy-eight participants (27.6%) with BG greater than 6.1 mmol/L were considered to be the abnormal glucose group. The z-scores of the cognitive scores between the normal and abnormal glucose groups were compared. Figure 1 shows the results after controlling for sex, age, education, and diabetes. There were significant differences in the RAVL-I (*p* = 0.020) and COWAT (*p* = 0.022) scores between the abnormal and normal glucose groups.

### Relationship between blood glucose, lipid profile, and cognitive performance in different APOE groups

The partial correlation analysis revealed that, in the APOE ε3 group, BG was negatively correlated with the cognitive scores from the RAVL-I (*r* = − 0.200, *p* = 0.005), COWAT (*r* = − 0.169, *p* = 0.019), RAVL-D (*r* = − 0.144, *p* = 0.040), and WAIS-BD (*r* = − 0.145, *p* = 0.045) tests. HDL was positively correlated with WMDS (*r* = 0.172, *p* = 0.017) and WAIS-BD (*r* = 0.203, *p* = 0.005) scores, and LDL was negatively correlated with MoCA scores (*r* = − 0.145, *p* = 0.044). In the APOE ε4 group, triglycerides were negatively correlated with WMVis-I scores (*r* = − 0.342, *p* = 0.025), and positively correlated with the WAIS-BD scores (*r* = 0.315, *p* = 0.039). In the APOE ε2group, there was no association between cognitive function and BG or lipid data.

In stepwise regression analyses in the different APOE groups, we used the MoCA, WMDS, RAVL-I, WMVis-I, COWAT, RAVL-D, and WAIS-BD scores as dependent variables, and the demographic variables, BG, and lipid data as independent variables. In the APOE ε3 group, BG was significantly associated with RAVL-I (*β* = − 0.141, *p* = 0.037), COWAT (*β* = − 0.140, *p* = 0.023), and WAIS-BD (*β* = − 0.173, *p* = 0.005) scores, and HDL was positive associated with WMDS (*β* = − 0.146, *p* = 0.011) scores. In the APOE ε4 group, higher BG was associated with worse performance on the WMVis-I test (*β* = − 0.301, *p* = 0.023).

### White matter hyperintensities in different APOE ε3-carrying BG groups

**Fig. 2:**
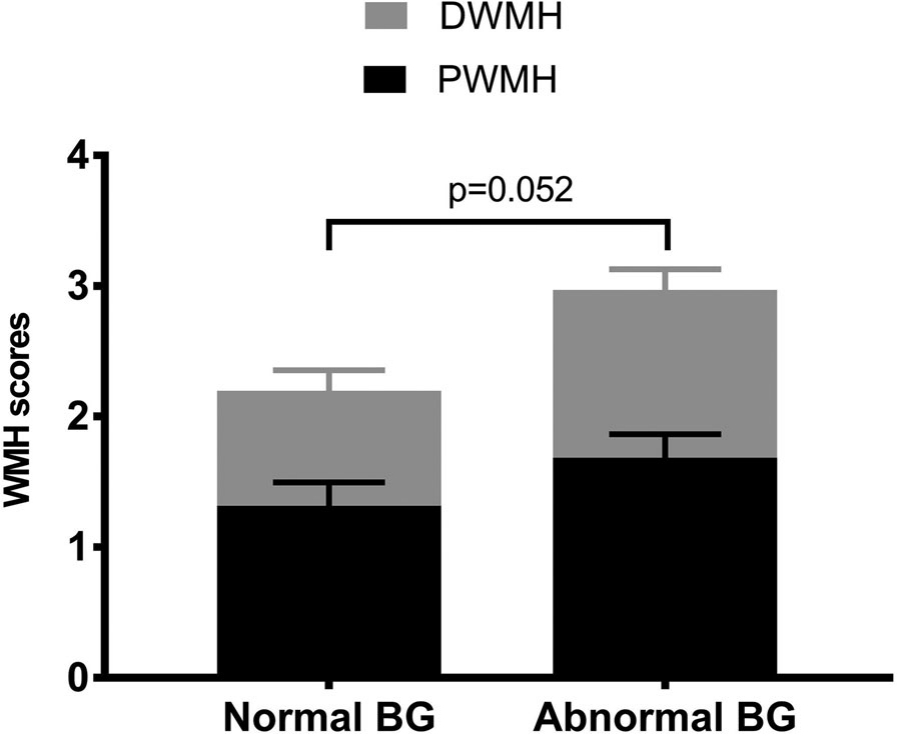
Differences in white matter hyperintensities between the normal and abnormal blood glucose groups. (PWMH: periventricular white matter hyperintensity, DWMH: deep white matter hyperintensity). The differences in the total WMH between the two groups approached significance (*t* = − 2.065, *p* = 0.052

There were 12 APOE ε3 carriers with abnormal BG, for whom WMH scores were available. We matched these with another 12 APOE ε3 carriers with normal BG in terms of age and sex. Eight participants in the abnormal BG group had a hypertension history, as compared to three in the normal BG group. We compared the periventricular WMH scores, deep WMH score, and total WMH score in the two BG groups, with adjustment for education and hypertension history. These results are shown in Fig. 2. The differences in the total WMH between the two groups approached significance (*t* = − 2.065, *p* = 0.052).

## Discussion

In this community-based, cross-sectional study, we found that cognitive decline in terms of immediate memory and executive function was associated with higher BG in non-demented, community-dwelling older adults. Perceptual reasoning was positively related to HDL. We also found that in the APOE ε3 group, elevated BG was associated with cognitive decline in terms of immediate memory, executive function, and perceptual reasoning. HDL was positively associated with executive function. In the APOE ε4 group, higher BG was also correlated with a decline in abstract reasoning, represented by WMVis-I performance. In the APOE ε2 group, BG was not related to cognitive function.

Some researchers have proposed that AD is a third type of diabetes that involves the central nervous system [25], and it is therefore interesting to determine the association between AD and glucose levels. Previous studies have indicated that diabetes or elevated BG exerts a specific influence on cognitive decline in dementia-free older adults [6, 26]. In this study, we found that elderly adults with higher BG had poorer cognitive performance in terms of immediate memory and executive function, consistent with reports from the Shanghai sampling study [5] and a multiethnic elderly cohort report [27]. Although many studies have reported that higher BG or diabetes was a risk factor of dementia, the results vary among different groups of people. Several reports from community-based studies have shown that individuals with high BG had lower scores in processing speed, executive function [8], and delayed memory [28]. Many cross-sectional studies have found no relationship between fasting blood glucose levels and cognitive function in people with type 2 diabetes without dementia [29]. The inconsistent results in terms of the different cognitive domains might be attributable to the age and sample of participants, as well as the cognitive assessment method. Our finding showed that high blood glucose was related to cognitive decline based on the RAVL-I and COWAT scores. RAVL-I not only assessed immediate memory but also attention and verbal reasoning. In addition to the executive function domain, COWAT also evaluated verbal ability. Since high BG is related to atrophy in the hippocampus [30], it is not surprising that high BG was related to working memory and verbal ability. In rat research, high BG has been shown to impair hippocampal synaptic plasticity [31]. BG was one component of the 7-point scale proposed by the American Heart Association. The Framingham cohort has shown that ideal cardiovascular health was negatively associated with vascular dementia, cognitive decline on visual memory and reasoning, and frontal brain atrophy [32]. It suggested that cardiovascular and metabolic health are closely linked to brain health.

Reitz et al. showed that dysglycemia was associated with lower performance in language, speed, and visuospatial function [27]; however, these associations were attenuated when adjustment was made for APOE ε4 and other vascular risk factors. This may indicate that the effects of elevated BG on cognitive impairment vary in the different APOE genotype groups. APOE ε3 was previously considered to be a neutral gene, but our previous study showed that APOE ε3/ε3 exhibited high blood glucose levels in Chinese non-demented older adults [16]. It is known that APOE ε3 is the most common genotype. In the current study, worse cognitive performance on immediate memory and executive function tests were significantly associated with higher BG levels, and these correlations were mainly manifested in the APOE ε3 group. This might suggest that APOE ε3 is a functional gene with different effects on cognition. In our study, an association was found between BG and COWAT in the APOE ε3 group, but not in the APOE ε4 group, which is inconsistent with earlier findings of an association of BG with verbal memory [33, 34]. We also found that APOE ε4 genotype carriers’ cognitive function in terms of abstract reasoning, but no other cognitive domains, was related to BG levels, which is partly consistent with the findings of a previous study [35, 36], but inconsistent with other research results [37]. This finding might be due to the small sample of APOE ε4 carriers in this study, and the age of the APOE ε4 group was younger than the other two groups. APOE ε4 is considered an established risk gene of late-onset AD. The main factors that cause cognitive decline in APOE ε4 carriers may not relate to BG. In the APOE ε2 group, BG was not associated with cognition and it may be that the protection offered by the ε2 allele offset the negative influence of BG on cognition.

Cholesterol plays an important role in cognitive function [38]. APOE genotypes are associated with cholesterol levels [39]. In our study, HDL was associated with the block design test scores in the APOE ε3 group and TG was associated with WMVis-I scores in the APOE ε4 group. This indicates that the APOE genotype might affect cognitive function via cholesterol or triglycerides.

Although some studies have demonstrated a significant association between diabetes and the severity of deep WMHs [40], others have shown no such association [41] or non-significant trends [5]. We showed that higher BG was associated with more severe deep WMH, but there were no statistically significant differences between the high (abnormal) fasting BG and normal fasting BG groups. A previous review had reported that diabetes was not associated with WMH progression [42]. However, the APOE ε4 risk allele was associated with higher WMH [43]. In our study, only a small sample had MRI data, and thus, the effect of higher BG on WMH in individuals with the APOE ε4 genotype carriers could not be addressed.

In addition to the small sample size, there are some other limitations in our study. This was a cross-sectional study, and the long-term effects of BG on cognition cannot be inferred. Furthermore, we only had data on fasting BG, which could not provide information on insulin sensitivity, and we did not assess hemoglobin A1c levels. In addition, in the statistical analyses, we did not exclude people with diabetes because individual history of this disease was self-reported by participants; however, this may have biased the results. Lastly, the ratings of white matter lesions were qualitatively assessed by a neurologist and not by quantifiable methods. Further research should consider expanding sample testing using MRI data so as to quantify any differences.

## Conclusion

Our results showed that older adults in a Chinese community who had high BG levels performed worse in various cognitive domains when they were APOE ε3/ε3 carriers, compared with the performance of those who carried the APOE ε4 or ε2 allele. High BG might also be related to WMH. The main clinical implication of our findings is that high BG also increases the risk of cognitive impairment, even among the “neutral” APOE ε3/ε3 genotype carriers. High BG may impact brain microvasculopathy in older adults. Thus, controlling BG in older individuals may reduce the risk of cognitive impairment. The need for interventions may be assessed according to the different APOE genotypes.

## Abbreviations

AD: Alzheimer’s disease
APOE: Apolipoprotein E
BG: Blood glucose
BMI: Body Mass Index
CLAS: China Longitudinal Ageing Study
COWAT: Controlled Word Association Test
DWMH: Deep white matter lesion hyperintensity
HDL-C: High destiny lipoprotein cholesterol
LDL-C: Low destiny lipoprotein cholesterol
MoCA: Montreal Cognitive Assessment
MRI: Magnetic resonance imaging
NTB: Neuropsychological Test Battery
PCR: Polymerase chain reaction
PWMH: Periventricular lesion hyperintensity
RAVL-D: Rey Auditory Verbal Learning Delayed
RAVL-I: Rey Auditory Verbal Learning-Immediate
SCID: Structured Clinical Interview for DSM-IV
TC: Total cholesterol
TG: Triglycerides
WAIS-BD: Wechsler Adults Intelligence Scale - Block Design
WAIS-PC: Wechsler Adults Intelligence Scale - Picture Completion
WMDS: Wechsler Memory-Digit Span
WMH: White matter hyperintensity
WMVis-I: Wechsler Memory Visual-Immediate
